# Quantification and Qualification of Bacteria Trapped in Chewed Gum

**DOI:** 10.1371/journal.pone.0117191

**Published:** 2015-01-20

**Authors:** Stefan W. Wessel, Henny C. van der Mei, David Morando, Anje M. Slomp, Betsy van de Belt-Gritter, Amarnath Maitra, Henk J. Busscher

**Affiliations:** 1 University of Groningen and University Medical Center Groningen, Department of Biomedical Engineering, Groningen, The Netherlands; 2 William Wrigley, Jr. Company, Chicago, Illinois, United States of America; University Hospital of the Albert-Ludwigs-University Freiburg, GERMANY

## Abstract

Chewing of gum contributes to the maintenance of oral health. Many oral diseases, including caries and periodontal disease, are caused by bacteria. However, it is unknown whether chewing of gum can remove bacteria from the oral cavity. Here, we hypothesize that chewing of gum can trap bacteria and remove them from the oral cavity. To test this hypothesis, we developed two methods to quantify numbers of bacteria trapped in chewed gum. In the first method, known numbers of bacteria were finger-chewed into gum and chewed gums were molded to standard dimensions, sonicated and plated to determine numbers of colony-forming-units incorporated, yielding calibration curves of colony-forming-units retrieved versus finger-chewed in. In a second method, calibration curves were created by finger-chewing known numbers of bacteria into gum and subsequently dissolving the gum in a mixture of chloroform and tris-ethylenediaminetetraacetic-acid (TE)-buffer. The TE-buffer was analyzed using quantitative Polymerase-Chain-Reaction (qPCR), yielding calibration curves of total numbers of bacteria *versus* finger-chewed in. Next, five volunteers were requested to chew gum up to 10 min after which numbers of colony-forming-units and total numbers of bacteria trapped in chewed gum were determined using the above methods. The qPCR method, involving both dead and live bacteria yielded higher numbers of retrieved bacteria than plating, involving only viable bacteria. Numbers of trapped bacteria were maximal during initial chewing after which a slow decrease over time up to 10 min was observed. Around 10^8^ bacteria were detected per gum piece depending on the method and gum considered. The number of species trapped in chewed gum increased with chewing time. Trapped bacteria were clearly visualized in chewed gum using scanning-electron-microscopy. Summarizing, using novel methods to quantify and qualify oral bacteria trapped in chewed gum, the hypothesis is confirmed that chewing of gum can trap and remove bacteria from the oral cavity.

## Introduction

Descriptions of the first use of chewing gum date back to the ancient Greek, who used tree resins from the mastic tree to quench thirst and refresh their breath. The first commercial chewing gum was not successfully marketed until the late 19^th^ century, when the rubbery tree sap of the Sapodilla tree formed the basis for gum manufacturing [1]. In the late 20^th^ century, chewing gum is not only regarded as a symbol of lifestyle, but also effects on cognitive performance, mood, alertness and appetite control have been reported [2–5]. Moreover, chewing gum has developed more and more towards an oral care and functional food product (“nutraceutical”), as it provides an easily applicable drug delivery vehicle with potential benefits for oral health [1]. High consumption rates, up to 2.5 kg per person per year, have made it into a billion dollar industry [6,7].

Most chewing gums consist of a mixture of food grade synthetic elastomers, like polyvinyl acetate or polyisobutylene, generally referred to as the gum-base [1]. Important requirements to gum-base materials are that they do not dissolve in the oral cavity and can be chewed for long periods of time without undergoing compositional and structural changes. In most commercially available chewing gums, the gum-base is supplemented with sweeteners, flavors and other bulking agents, while nowadays sugar is frequently replaced by artificial sweeteners such as sorbitol, xylitol or mannitol [6,7].

The inclusion of xylitol and other artificial sweeteners has been described to reduce the formation of oral biofilms on teeth [8,9]. Oral biofilms are causative to the world’s most wide-spread infectious diseases, namely dental caries and periodontal disease [10]. Caries arises from an unbalance between naturally occurring de- and remineralization of dental enamel. Demineralization occurs when the pH of oral biofilm drops below 5.5 [11] due to the fermentation of carbohydrates by specific bacterial strains in oral biofilms on teeth. Most artificial sugars are not or barely fermented by oral bacteria and therewith do not lower the pH [12]. Moreover, chewing gum yields enhanced mastication that stimulates salivation, which clears fermentable carbohydrates, dislodges loosely bound oral bacteria from oral surfaces [13] and increases the concentrations of calcium and phosphates in the oral cavity required for remineralization [14]. Fluorides have been added to commercial gums to prevent enamel demineralization and stimulate remineralization [15]. It is tempting to regard the chewing of gum as an addendum to daily oral hygiene procedures, especially since most people are unable to maintain a level of oral biofilm control required to prevent disease through daily toothbrushing and other conventional oral hygiene measures. This has led to the incorporation of antimicrobials like chlorhexidine [16] and herbal extracts [17] to chewing gums and gums have indeed been demonstrated successful in preventing re-growth of oral biofilm [18]. It is also known that chewing of gum aids in the removal of interdental debris [19]. To increase the cleaning power of chewing gum, detergents like polyphosphates [20] have been added to gums. However, it is unclear whether chewing of gum itself will actually remove bacteria from the oral cavity. Especially the preferential removal in sizeable numbers of disease-causing microorganisms like acid-producing *Streptococcus mutans* or species that are regarded as initial colonizers of tooth surfaces by chewing gum would turn chewing gum into a valuable addendum to daily oral hygiene.

Therefore, the aim of this study is firstly to develop methods to quantify the number of bacteria that are trapped into a gum after chewing, and secondly to qualitatively determine the bacterial composition of bacteria trapped in chewed gums. The first method is based on measuring the number of colony-forming units (CFUs) that can be retrieved from pieces of gum, chewed by different volunteers. The method relies on finger-chewing known numbers of different oral bacterial strains into commercially available spearmint gums and retrieving bacteria from the gums by sonication followed by agar-plating of the bacterial suspension to yield a calibration curve. By comparing it to the number of bacteria retrieved from pieces of gum chewed by volunteers, the number of CFUs trapped in pieces of chewed gum can be calculated. In the second method, pieces of chewed gum are dissolved and the amount of bacterial genomic DNA is quantitated using quantitative Polymerase-Chain-Reaction (qPCR) and converted to numbers of bacteria trapped in the chewed gums using a calibration curve, also obtained by finger-chewing. The composition of the different bacterial species trapped in chewed gum was compared with the composition of the salivary microbiome and the microbiome adhering to teeth using Denaturing Gradient Gel Electrophoresis (DGGE). Finally, we demonstrate bacterial presence in chewed gum using Scanning Electron Microscopy (SEM).

## Materials and Methods

### Chewing Gum

Two commercially available spearmint chewing gums were used in this study: Gum A – (commercially available spearmint gum, 1.5 g tabs). Composition in descending order of predominance by weight: Sorbitol, gum base, glycerol. Natural and artificial flavors; less than 2% of: Hydrogenated starch hydrolysate, aspartame, mannitol, acesulfame K, soy lecithin, xylitol, beta-carotene, blue 1 lake and butylated hydroxytoluene.

Gum B – (commercially available spearmint gum, 1.5 g tabs.). Composition in descending order of predominance by weight: Sorbitol, gum base, glycerin, mannitol, xylitol. Natural and artificial flavors; less than 2% of: Acesulfame K, aspartame, butylated hydroxytoluene, blue 1 lake, soy lecithin and yellow 5 lake. Both gums were similarly hydrophobic with water contact angles on sectioned pieces of gum of 69 and 74 degrees for gum A and B, respectively.

### Method 1: Enumeration of Bacteria Trapped in Chewed Gums using Sonication of Gum Molded to Standard Dimensions

#### Basics of the Method and Preparation of a Calibration Curve

In this method, four different bacterial strains were used for the preparation of a calibration curve that relates the numbers of CFUs retrieved from a piece of gum to the numbers of CFUs incorporated in the gum for coccus-shaped *Streptococcus oralis* J22, *Streptococcus mutans* ATCC 25175, *Streptococcus mitis* ATCC 9811 and rod-shaped *Actinomyces naeslundii* T14V-J1. *S. oralis* and *A. naeslundii* are considered initial colonizers of tooth surfaces *in vivo* [21,22], while *S. mutans* is causative to dental caries [23] and *S. mitis* is an abundantly present species in the oral cavity [24]. Streptococci were grown aerobically in Todd Hewitt Broth (THB) at 37°C and actinomyces anaerobically in Schaedler broth. Bacteria were first grown on THB agar or blood agar plates from a frozen stock in dimethylsulfoxide for 24 h after which one colony was inoculated in 10 ml of the appropriate culture medium and incubated for 24 h. A main culture was prepared with a 1:10 dilution in fresh medium for 16 h. Main cultures were sonicated for 1 × 10 s at 30 W (Vibra Cell model 375, Sonics and Materials Inc., Danbury, CT, USA) to suspend bacterial aggregates. The bacterial concentration was determined using the Bürker Türk counting chamber, while percentage viability of the suspended bacteria was determined after serial dilution and agar-plating. Next, concentrations were adjusted to 10^4^, 10^5^, 10^7^ and 10^9^ bacteria per ml. Since viability of the cultures was near 100%, these numbers are equivalent to 4, 5, 7 and 9 log-units of CFUs per ml.

For each strain, known numbers of CFUs were finger-chewed into gum pieces by adding 1.5 g chewing gum together with 200 μl of a bacterial suspension into the finger of a sterile latex glove (Powder-Free Latex Examination Gloves, VWR international, Radnor, USA). Next, bacteria were finger-chewed into the gum in a water bath at 37°C for 5 min. After finger-chewing, the gum was removed from the glove, dipped once in 10 ml sterile water and put into a Teflon mold (15 × 15 × 1 mm) with a sterile pair of tweezers to create reproducible gum dimensions (15 × 15 × 4 mm) and surface area (690 mm^2^). Subsequently, the gum was inserted in sterile polystyrene cups with 5 ml filter sterile Reduced Transport Fluid (RTF) [25]. Bacteria were removed from the gum surface layer by sonication for 60 s in a water bath sonicator (ELMA Transsonic TP690, Elma GmbH & Co, Germany). Sonication times up to 60 s did not affect bacterial viability [26,27]. Finally, the resulting suspension was serially diluted, plated on THB agar or blood agar plates (Blood agar base no. 2, 40 g/l, hemin 5 mg/l, menadion 1 mg/l, sheep blood 50 ml/l) and incubated at 37°C for 48 h after which the number of CFUs retrieved were counted. Accordingly, since different numbers of bacteria were finger-chewed into the gums, a calibration curve was made of the numbers of CFUs retrieved from each gum for the different bacterial strains *versus* the numbers of CFUs finger-chewed into the gum. To account for possible loss of bacteria due to adhesion to the inner surface of the glove, the glove finger was turned inside out after removal of the gum and sonicated in 10 ml filter sterile RTF for 60 s and serial dilutions plated on agar plates as described above after which the number of CFUs lost were determined. Similarly, the water in which the finger-chewed gums were dipped (see above) was analyzed for bacterial losses. Calibration curves were made in triplicate for each chewing gum and bacterial strain.

#### Application of the Method in Human Volunteers

Volunteers included in this study were five healthy members of the department of Biomedical Engineering (1 male, 4 females, aged 27 to 56 years). All experiments were performed according to the rules as set out by the Medical Ethics Committee of the University Medical Center Groningen, and they approved this study (approval METc 2011/330). Volunteers gave their written informed consent. Inclusion criteria described that all volunteers should be in good health and have at least 16 natural elements. Exclusion criteria were the use of antibiotics or mouth rinses in the month prior to the study or the use of antibiotics, mouth rinses and additional chewing gum during the study. Furthermore, volunteers were requested to brush their teeth twice a day, according to their habitual routines.

On separate days, volunteers were asked to chew 1.5 g (one serving size) of each chewing gum once a day at the same time for 0.5, 1, 3, 5 or 10 min according to their own personal routine without specific instructions for chewing. Chewing time and gum types (A or B) were randomly assigned to the volunteers over the experimental period. After chewing, the gum was spit in a polystyrene cup with 10 ml sterile water, after which the chewed gum was put into the Teflon mold and sonicated, as described above. Resulting suspensions were serial diluted, agar-plated and the numbers of CFUs were determined after incubation for 7 days at 37°C under anaerobic conditions (5% H_2_, 10% CO_2_, 85% N_2_) (Concept 400 anaerobic workstation, Ruskinn Technology Ltd., Pencoed, UK). Finally, the numbers of CFUs retrieved from the gums after different chewing times and for both types of gum were converted to the total number of CFUs trapped in chewed gums using the calibration curve obtained from finger-chewing known numbers of bacteria into the gums. Note that this requires the assumption that bacterial viablility is equally maintained in finger-chewed gum as in gum chewed by volunteers. All experiments were carried out in duplicate for each volunteer, gum type and time point.

### Method 2: Enumeration of Bacteria Trapped in Chewed Gums using qPCR and Microbial Composition

#### Basics of the Method and Preparation of a Calibration Curve

Similar to method 1, a calibration curve was made by finger-chewing known numbers of *S. oralis* J22, *S. mutans* ATCC 25175, *S. mitis* ATCC 9811 or *A. naeslundii* T14V-J1 in the different spearmint gums. Bacterial concentrations were adjusted using the Bürker Türk counting chamber to 10^7^, 10^9^ and 10^10^ bacteria per ml, in which the latter concentration was achieved by centrifugation (5 min, 5000 g at 10°C). After finger-chewing as described above, the gum was removed from the glove, dipped once in 10 ml sterile water and subsequently dissolved in a mixture of 5 ml chloroform (67-66-3, Fisher Scientific, Waltham, USA) and 3 ml tris-ethylenediaminetetraacetic-acid (TE) buffer (AM9849, Ambion—LifeTechnologies, Carlsbad, USA) in a sterile centrifuge tube. The gum was dissolved in 45 min by shaking horizontally. The resulting suspension was centrifuged for 10 min at 1500 g to remove large particles and gum base from the aqueous TE buffer top layer.

For qPCR, 17.5 μl master mix was used for every sample consisting of 10 μl PCR—mix (iQ5 SYBR Green Supermix, Bio-rad, Hercules, USA), 5 μl DNA free water (95284, Sigma, St. Louis MO, USA) and 2.5 μl primer mix (300 nM). To amplify the universal V3 region of the 16S rRNA gene in all samples F357-GC was used as the forward primer and R-518 [28] as the reverse primer. In a 384-well PCR plate (HSP-3805, Bio-rad, Hercules, USA ), 2.5 μl of sample dilutions (1×, 10×, 100×), taken from the centrifuged aqueous TE buffer top layer, was mixed with 17.5 μl of master mix. Subsequently, a qPCR was performed on a thermocycler (CFX384, Bio-rad, Hercules, USA), according to a 3 step amplification (95.0°C for 45 s, 58.0°C for 45 s, 72°C for 60 s) of 39 cycles. A calibration curve was obtained by relating threshold cycle (Ct) at fixed relative fluorescence units to the number of bacteria chewed-in the gum [29,30]. Calibration curves were obtained for both gums in triplicate for all four bacterial strains. DNA free water and a piece of unchewed gum, dissolved as described above, were used as negative controls.

#### Application of the Method in Human Volunteers

Five healthy members of the department of Biomedical Engineering chewed each type of chewing gum, as described above. Chewed gum was spit in a polystyrene cup with 10 ml sterile water after which the gum was dissolved in a sterile centrifuge tube with the mixture of chloroform and TE buffer. After centrifugation, qPCR was performed using the aqueous TE buffer top layer (see above). The total number of bacteria trapped in the gum was determined using the calibration curve. Part of each dissolved gum TE-buffer sample was stored in −80°C for later DGGE analysis.

#### Determination of the Bacterial Composition using DGGE

The composition of the different species trapped in pieces of chewed gum was determined using DGGE and compared to the bacterial compositions of the planktonic, salivary microbiome and the microbiome adhering to tooth surfaces. After 10 min of chewing, volunteers were asked to donate 1 ml of unstimulated saliva and collect oral biofilm from their entire dentition using a cotton swab and a sterile hook in 1 ml RTF. Both saliva and biofilm samples were centrifuged at 18000 g for 5 min (Eppendorf Centrifuge 5417R, Hamburg, Germany), DNA was isolated [31], after which the samples were resuspended in 50 μl TE buffer.

The DNA concentration of saliva, biofilm and dissolved gum samples were measured with the Nanodrop Spectrophotometer (ND-110, NanoDrop Technologies Inc., Wilmington, DE, USA). A PCR was performed with 100 ng DNA using the primers and amplification program as described above. The products of the PCR were applied on a polyacrylamide gel (8% w/v) in 0.5 TAE buffer (20 mM Tris acetate, 10 mM sodium acetate, 0.5 mM EDTA, pH 8.3). Using a 100% stock solution (7 M urea, 37% formamide) a denaturing gradient was made with the range of 30–80%. A stacking gel without denaturant was added on top and equal amounts of sample were applied to the gel. Electrophoresis was performed overnight at 60°C and 120 V. Silver nitrate solution (0.2% AgNO_3_) was used until maximal staining intensity was reached.

Gels were scanned and transferred to analysis software BioNumerics (v7.1 Applied Maths, Sint-Martens-Latem, Belgium). Gels were normalized to reference markers that were added to every gel. Presence of a band on the gel was taken as the presence of a bacterial species or strain in the sample. The similarity of bands was determined according to the band-based matching module in the software (0.5% optimization, 1% band tolerance).

### Scanning Electron Microscopy

In order to visualize bacteria trapped in chewed gum, a 5 min chewed gum piece was spit into liquid nitrogen, kept immersed for 2 min and broken into multiple pieces, which were subsequently examined in a SEM (JEOL JSM-6301F, Akishima, Japan). Gum pieces were fixed directly for 24 h in 2.0% glutaraldehyde at 4.0°C, washed with 0.1 M cacodylate buffer and incubated for 1 h in 1.0% OsO_4_ in 0.1 M cacodylate buffer at room temperature. After washing with water, samples were dehydrated with an ethanol series (30, 50 and 70%) each for 15 min and 3 times 30 min with 100% ethanol. Fracture surfaces of the chewed gum were examined for the presence of bacteria at a magnification of 7.500× with an acceleration voltage of 2.0 kV and 39.0 mm working distance.

### Statistics

Data was evaluated for normality using Shapiro-Wilk and Kolmogorov-Smirnov test (p < 0.05) and in case of a normal distribution equality of means was tested using an ANOVA followed by Tukey-HSD post hoc test (p < 0.05). In case no normal distribution of data was observed, a non-parametric Kruskal-Wallis test was used (p < 0.05). SPSS v20.0 (IBM Corp., Armonk, USA) to conduct all statistical analysis.

## Results

Bacteria of the four different strains were finger-chewed into the two different types of chewing gums in order to obtain a relation between the number of bacteria trapped in a gum piece and the number of CFUs or total bacteria that can be retrieved from a gum by agar-plating or qPCR, respectively. On average, 0.05 log-units of CFUs were lost due to adhesion to the surface of the glove in which gums were finger-chewed, while *A. naeslundii* adhered in slightly higher numbers to the glove surface than streptococcal strains. Bacterial losses due to dipping the finger-chewed gum pieces in water were much smaller and amounted on average 0.004 log-units of CFUs.

**Figure 1:**
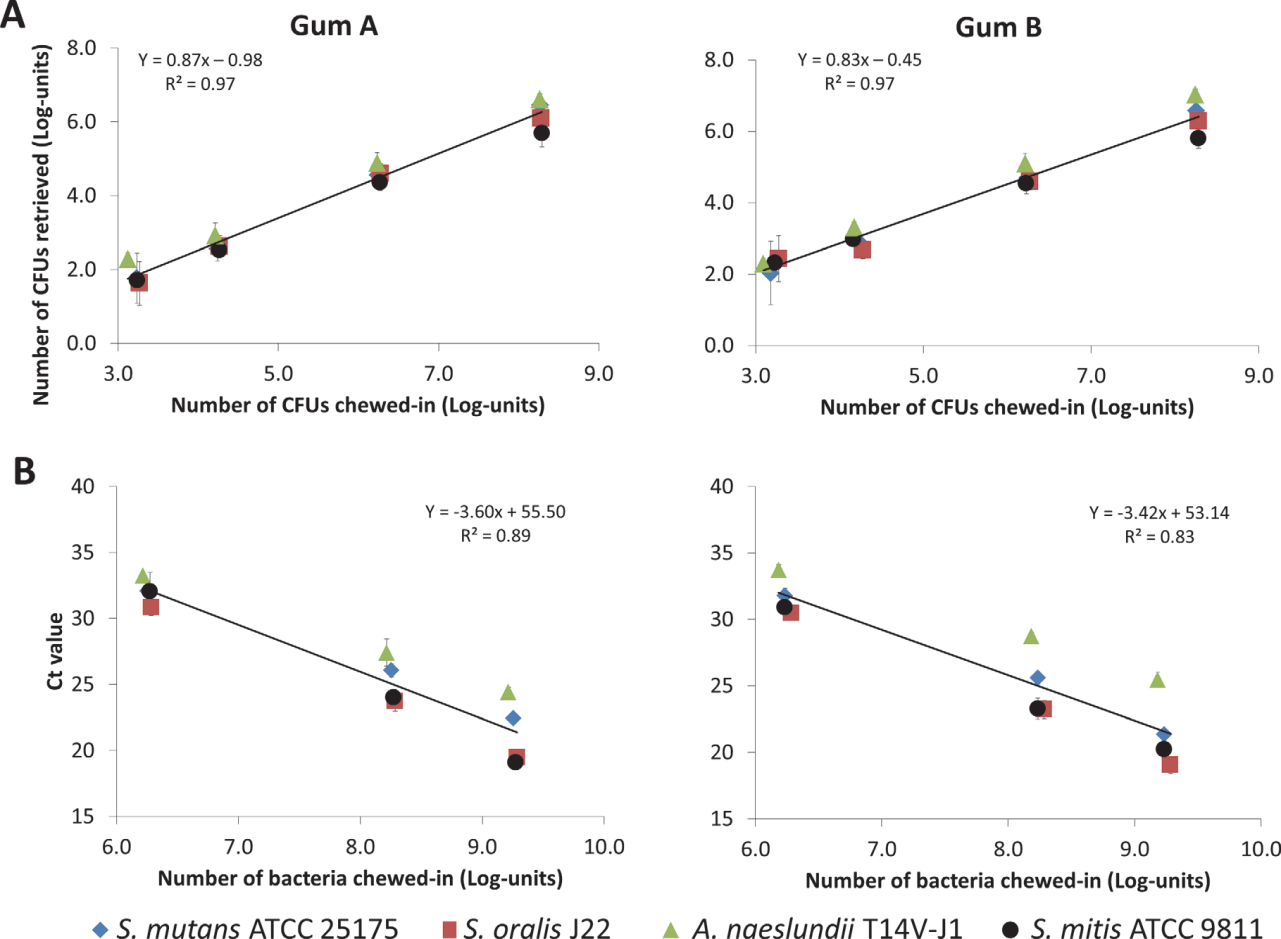
Calibration curves for bacterial trapping in finger-chewed gums. Calibration curves for bacterial trapping after finger-chewing known numbers of bacteria into gum. Results are obtained from three independent experiments with separately cultured bacteria. Data are corrected for losses of bacteria due to adhesion to the glove-finger and during water rinsing. Error bars denote the standard deviation over triplicate experiments and linear relations are presented by the equations with their corresponding correlation coefficients. A. The number of CFUs retrieved as a function of the numbers of CFUs finger-chewed in a gum piece for the four different bacterial strains, obtained by sonication of chewed pieces of gum, molded into a standard dimension and followed by sonication and agar-plating. B. The number of threshold cycles (Ct) at fixed relative fluorescence units as a function of the total number of bacteria finger-chewed in a gum piece for the four different bacterial strains, obtained after dissolving the gum in chloroform and TE buffer and performing qPCR.

Accounting for these losses, linear relations were obtained for both methods (Fig. 1). For CFUs, the calibration lines were independent of the gum type involved. Lines were generally independent of the bacterial strains involved, apart from a small but statistically significant difference (p < 0.05) between *A. naeslundii* and *S. mitis* at the highest bacterial concentration (Fig. 1A). As sonication can only release bacteria trapped in a gum from the outer surface, the number of bacteria retrieved was roughly 1.5 log-units less than chewed-in. The qPCR method yielded small but statistically significant differences (p < 0.05) in Ct values for the different bacterial strains (Fig. 1B). However, neglecting these strain-related differences, average linear calibration lines could be obtained that were independent of the gum type involved.

**Figure 2:**
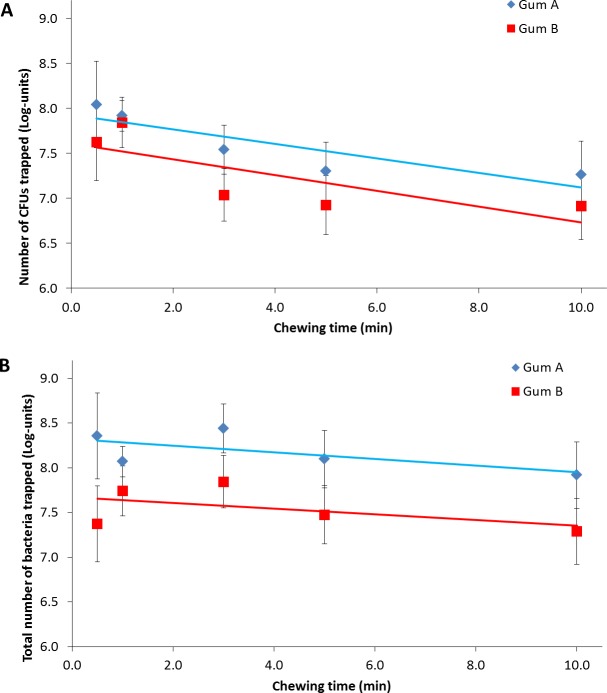
Bacteria trapped in two different types of spearmint gums chewed by human volunteers as function of time. The number of bacteria trapped in chewed gums for two types of spearmint gums as a function of the chewing time. Error bars denote the standard deviation over a group of five volunteers, with each volunteer having chewed the same gum twice for all time points. A. CFUs trapped per gum piece obtained after molding, sonication and agar-plating. B. Total number of bacteria trapped per gum piece obtained after dissolving the gum and performing qPCR.

Next, volunteers were asked to chew the two types of chewing gums for varying amounts of time up to 10 min and the number of bacteria chewed-in was determined in terms of CFUs after sonication and agar-plating or in terms the total number of bacteria, as obtained after dissolving the gum and performing qPCR on bacterial DNA. Agar plating indicates that most CFUs are trapped (approximately 7.8 log-units) within the first minute, regardless of the gum involved, while approximately 1 log-unit less CFUs remained trapped in a gum piece after prolonged chewing (Fig. 2A). qPCR yields higher numbers of bacteria retrieved than agar-plating (Fig. 2B), but displays only a minor decrease in total number of bacteria trapped in time for both types of chewing gums.

**Figure 3:**
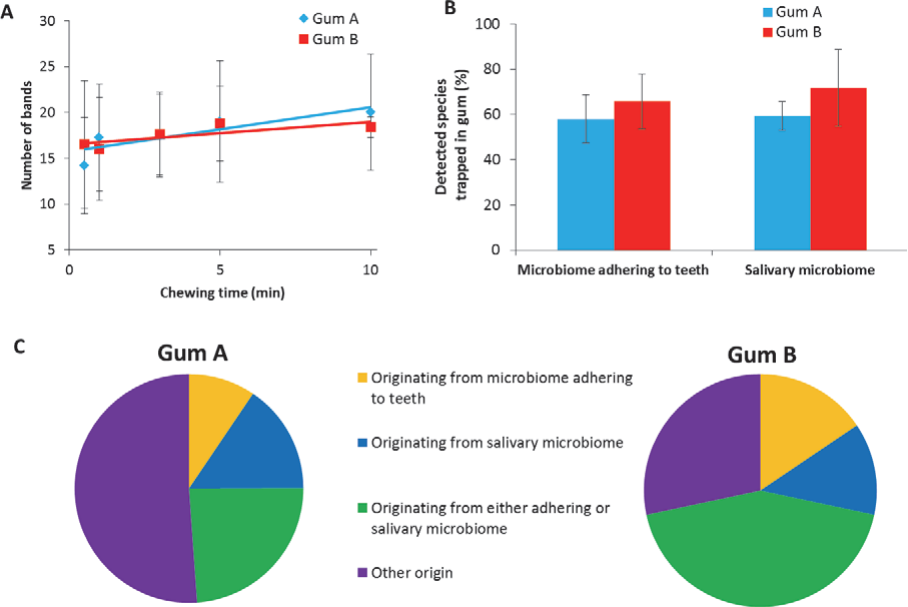
Diversity of bacterial strains and species trapped in chewed gum in comparison with the bacterial diversity in the salivary microbiome and the micobiome adhering to tooth surfaces. A. The number of bands in DGGE gels in bacterial DNA obtained from pieces of chewed gum as a function of the chewing time. Error bars denote the standard deviation over a group of five volunteers. No statistically significant differences were observed. B. Percentage of species detected in the microbiome adhering to tooth surfaces or in the salivary microbiome relative to the number of species found in chewed gum (10 min of chewing) set at 100%. Error bars denote the standard deviation over a group of five volunteers. No statistically significant differences were observed. C. Percentage of species found in chewed gum based on origin, i.e. found in chewed gum and the adhering microbiome, chewed gum and the salivary microbiome and found in gum and both microbiomes. The category “other origin” indicates species that were solely found in chewed gum and below detection in the salivary and in the adhering microbiome.

The number of species detected in chewed gum increases with increasing chewing time for both types of chewing gums (Fig. 3A), while after 10 min of chewing 50–70% of the detected species in the salivary and adhering microbiome are ultimately detected in the chewed gum piece (Fig. 3B). A more elaborate analysis of the origin of bacterial species found in chewed gum indicated that 9% and 16% of the species found in chewed gum were solely detected in the adhering oral microbiome for gum A and B, respectively, while a relatively similar percentage of approximately 15% of the detected species chewed-in were solely found in the salivary microbiome (Fig. 3C). Remaining percentages of species found in chewed gum could either be attributed to the salivary or the adhering microbiome or their origin could not be detected, suggesting the tongue, gums or oral mucosal surfaces as an origin.

**Figure 4:**
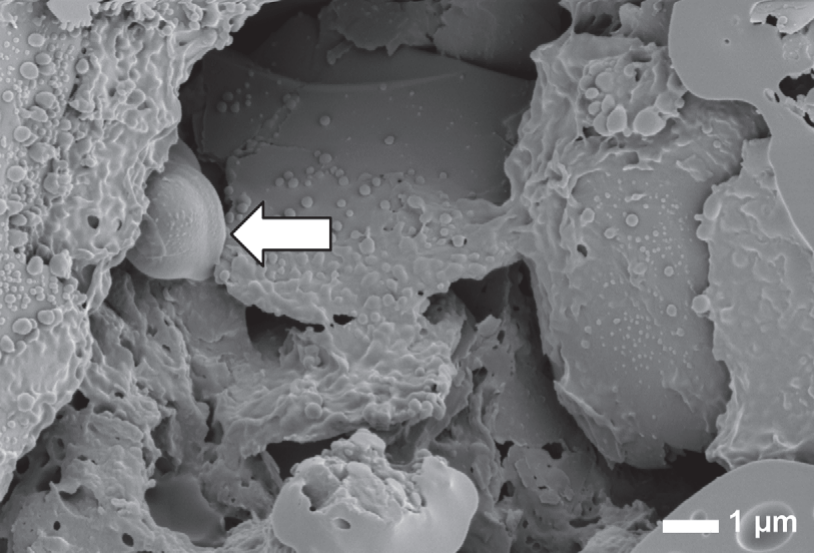
SEM visualization of bacteria trapped in a piece of chewed gum. Scanning electron micrograph of a bacterium (indicated by white arrow) trapped in a chewed gum piece of gum A. The scale bar indicates 1 μm.

Considering the numbers of bacteria found in chewed gum and the field of view and depth of focus of SEM, it can be appreciated that microscopic imaging of trapped bacteria in chewed gum is like looking for a needle in a haystack. Yet after extensive searching, a scanning electron micrograph could be taken of a chewed gum piece showing an open and porous structure (Fig. 4) in which trapped bacteria can be observed as direct evidence of the ability of chewing gum to trap bacteria during chewing.

## Discussion

In this paper we provide evidence that bacteria are trapped inside gum pieces chewed by human volunteers and therewith may contribute to the maintenance of oral health. The number of bacteria trapped in chewed gums were determined using two distinctly different methods. Finger-chewing and subsequent sonication and agar-plating demonstrated that approximately 1–1.5 log-units less than the number of bacteria chewed-in could be retrieved, regardless of the type of gum or bacterial strain involved, i.e. coccus- or rod-shaped microorganisms (Fig. 1A). Although this recovery is confined to the surface layer of the gums amenable to sonic removal of chewed-in bacteria and therefore relatively low, it allows to culture the bacteria retrieved and express them in terms of CFUs. Compared to qPCR, which requires chemical dissolution of the gum and bacterial lysis to determine the presence of genomic DNA from bacteria trapped in chewed gums, agar-plating yields lower numbers of trapped bacteria, likely because qPCR includes both dead and live bacteria [32] while agar-plating only reports viable ones. Whereas agar plating yielded results that were independent of the bacterial strain involved, Ct values obtained in qPCR were somewhat strain-dependent (Fig. 1B), possibly due to differences in efficacy of lysis of the different strains and the relative efficiencies of the primer pairs used. However, since calibration curves are applied to bacterial samples of unknown composition, the small strain-dependent differences in Ct values were neglected and average calibration curves were calculated and employed.

Both methods indicate a slow but significant decrease in bacterial trapping with increasing chewing time in human volunteers after an initial maximum, regardless of the type of gum involved. Whereas the initial gum bases are thus most adhesive to oral bacteria (Fig. 2) continued chewing changes the structure of the gums, decreasing the hardness of the gum due to uptake of salivary components [33] and release of water soluble components. This presumably affects the adhesion of bacteria to the gum [34], causing a release of initially trapped, more weakly adhering bacteria from the gum. Such a change in composition of trapped bacteria is supported by the observation that the diversity of species trapped in chewed gum increases with chewing time (Fig. 3A).

Despite an increasing diversity in species developing over time in chewed gums, there is a gradual decrease in the number of bacteria trapped in chewed gum over time. This can be attributed to a decrease in bacterial concentration in saliva during chewing, shown in earlier reports [13]. However, alternative explanations exist as well, especially since this decrease is far more prominent for the numbers of CFUs retrieved than for the total numbers of bacteria found by qPCR in chewed gum. This difference in decrease suggests that bacteria are killed during their entrapment in the gum by sweeteners like xylitol, food preservatives or flavoring agents like spearmint and peppermint, which are reported to have antimicrobial properties [9,35–37].

Numbers of bacteria trapped in a chewed piece of gum amount around 10^8^ depending on the time of chewing and retrieval method. Although this number may be considered low, it shows that when gum is chewed on a daily basis, it may contribute on the long-term to reduce the bacterial load in the oral cavity, which is supported by observations that long-term studies on the use of chewing gum cause a reduction in the amount of oral biofilm [38]. Bacteria trapped in chewed gum can originate either from the salivary microbiome or the adhering microbiome on teeth, but also from the tongue, gums or oral mucosal surfaces from which we did not sample. No DNA was detected in unchewed gum pieces. Saliva harbors up to 10^9^ microorganisms per ml before chewing [11,39]. Assuming a volume of saliva of around 1 ml in the oral cavity, our results indicate that chewing of one piece of gum removes around 10% of the oral microbial load in saliva. However, as our DGGE results pointed out, saliva does not necessarily have to be the source of the bacteria found trapped in chewed gum. Making the alternative assumption that all bacteria trapped in chewed gum come from the adhering microbiome, we can place this number in further perspective by comparing it to the number of bacteria removed by toothbrushing. Using a new, clean toothbrush without any toothpaste reportedly removes around 10^8^ CFUs per brush [39,40], which would put chewing of gum on par with the mechanical action of a toothbrush. Moreover, also the mechanical action of floss wire removes a comparable number of bacteria from the oral cavity than does chewing of a single piece of gum, as we established in a simple pilot involving 3 human volunteers who used 5 cm of floss wire (unpublished). Chewing however, does not necessarily remove bacteria from the same sites of the dentition as does brushing or flossing, therefore its results may be noticeable on a more long-term than those of brushing or flossing [7,19,41].

Our findings that chewing of gum removes bacteria from the oral cavity, may promote the development of gum that selectively removes specific disease-related bacteria from the human oral cavity, for instance by using porous type calcium carbonate [42]. It is known that the key to oral health is a balanced and diverse composition of the oral microbiome, although the exact composition of what is tentatively called “the oral microbiome at health” is not known. Removal of specific pathogens however, is directly in line with the general notion arising in dentistry that oral diseases develop when the oral microbiome shifts its composition into a less diverse direction [43]. In this respect, a gradual removal of bacteria from the oral cavity through regular removal of low numbers of pathogens by chewing gum is preferable to sudden ecological shifts that can change the relationship between the oral microbiome and the host as another potential cause of disease [43].
